# Cerebral small vessel disease, cardiovascular risk factors, and future walking speed in old age: a population-based cohort study

**DOI:** 10.1186/s12883-021-02529-6

**Published:** 2021-12-24

**Authors:** Emerald G. Heiland, Anna-Karin Welmer, Grégoria Kalpouzos, Anna Laveskog, Rui Wang, Chengxuan Qiu

**Affiliations:** 1 Department of Surgical Sciences, Medical Epidemiology, Uppsala University, Dag Hammarskjölds väg 14B, 75 185 Uppsala, Sweden; 2 Aging Research Center, Department of Neurobiology, Care Sciences and Society, Karolinska Institutet-Stockholm University, Widerströmska Huset, Tomtebodavägen 18A, 171 65 Solna, Sweden; 3 Department of Physical Activity and Health, The Swedish School of Sport and Health Sciences (GIH), Lidingövägen 1, 114 86 Stockholm, Sweden; 4 Stockholm Gerontology Research Center, Sveavägen 155, 113 46 Stockholm, Sweden; 5 Women’s Health and Allied Health Professionals Theme, Medical Unit Medical Psychology, Karolinska University Hospital, Stockholm, Sweden; 6 Division of Physiotherapy, Department of Neurobiology, Care Sciences and Society, Karolinska Institutet, Alfred Nobels allé 23, 141 83 Huddinge, Sweden; 7 Division of Neuro, Department of Clinical Neuroscience, Karolinska Institutet, Tomtebodavägen 18A, 171 65 Solna, Sweden; 8 Department of Neuroradiology, Karolinska University Hospital, Eugeniavägen 3, 171 76 Solna, Sweden

**Keywords:** Cerebral small vessel disease, Walking speed, Physical function, Cardiovascular risk factors, Magnetic resonance imaging (MRI), Population-based cohort study

## Abstract

**Background:**

The purpose of this study was to examine the associations between combined and individual cerebral small vessel disease (cSVD) markers on future walking speed over 9 years; and to explore whether these associations varied by the presence of cardiovascular risk factors (CRFs).

**Methods:**

This population-based cohort study included 331 adults, aged ≥60 years, without limitation in walking speed (≥0.8 m/s). At baseline, cSVD markers, including white matter hyperintensities (WMH), lacunes, and perivascular spaces (PVS), were assessed on magnetic resonance imaging. The modifiable CRFs (physical inactivity, heavy alcohol consumption, smoking, hypertension, high total cholesterol, diabetes, and overweight/obese) were combined into a score. The association between baseline cSVD markers and the decline in walking speed was examined using linear mixed-effects models, whereas Cox proportional hazards models were used to estimate the association with walking speed limitation (defined as < 0.8 m/s) over the follow-up.

**Results:**

Over the follow-up period, 76 (23.0%) persons developed walking speed limitation. Participants in the highest tertile of the combined cSVD marker score had a hazard ratio (HR) of 3.78 (95% confidence interval [CI] 1.70-8.45) for walking speed limitation compared with people in the lowest score tertile, even after adjusting for socio-demographics, CRFs, cognitive function, and chronic conditions. When investigating the cSVD markers individually, having the highest burden of WMH was associated with a significantly faster decline in walking speed (β coefficient − 0.020; 95% CI -0.035-0.004) and a greater HR of walking speed limitation (HR 2.78; 95% CI 1.31-5.89) compared with having the lowest WMH burden. Similar results were obtained for the highest tertile of PVS (HR 2.13; 95% CI 1.04-4.36). Lacunes were associated with walking speed limitation, but only in men. Having ≥4 CRFs and high WMH volume simultaneously, showed a greater risk of walking speed limitation compared with having ≥4 CRFs and low WMH burden. CRFs did not modify the associations between lacunes or PVS and walking speed.

**Conclusions:**

Combined cSVD markers strongly predict walking speed limitation in healthy older adults, independent of cognitive function, with WMH and PVS being the strongest contributors. Improving cardiovascular health may help to mitigate the negative effects of WMH on future walking speed.

**Supplementary Information:**

The online version contains supplementary material available at 10.1186/s12883-021-02529-6.

## Introduction

Walking speed is a fundamental physical function for performing everyday activities of daily living, whereas impairment in this function is a strong predictor of dementia, disability, institutionalisation, and death [1–3]. Cerebral microvascular lesions are characterised on magnetic resonance imaging (MRI) as markers of cerebral small vessel disease (cSVD) [4, 5]. These lesions are increasingly common as people age, and are indicative of subclinical abnormalities [6]. Evidence exists that a high burden of cSVD markers is associated with subsequent cognitive impairment among healthy older adults, and can accelerate cognitive decline [7]. Of the cSVD markers, increasing volume of white matter hyperintensities (WMH) has been repeatedly shown to be associated with cognitive impairment and dementia [8]. Moreover, WMH have also been linked with impairment in gait parameters [9–12]. A previous study has shown that clinical markers, such as poor walking speed together with MRI markers of cSVD can predict 8-year mortality [13]. However, WMH may only explain part of the decline in walking speed, as other cSVD markers, which develop concomitantly, may also be associated walking speed impairment and its progressive decline, as demonstrated similarly for cognitive deterioration [7]. In addition, various cSVD markers may represent different stages of pathology although of similar vascular origin.

Apart from WMH, other cSVD markers, such as lacunes and perivascular spaces (PVS) have not been previously investigated in relation to future walking speed, and their combined effect with WMH on walking speed impairment has only been previously examined cross-sectionally [9, 12, 14]. Understanding of the association between these imaging markers and prognosis of walking speed can be beneficial clinically and for public health in order to identify older persons at greater risk of poor functional outcomes, so that interventions can be implemented early. This can help older adults to remain independent longer and maintain a good quality of life.

Moreover, age, sex, and cardiovascular risk factors (CRFs) may modify the relationship between the cSVD markers and incipient walking speed impairment [2, 15]. As cSVD development is correlated with an underlying vascular pathology, a favourable lifestyle may reduce the risk of cSVD progression. In fact, previous findings have revealed that individual and combined CRFs are associated with a greater risk of developing walking speed impairment in older adults [2].

Thus, in this population-based cohort study of older adults, the primary aim was to examine the associations of single cSVD markers and their collective burden with walking speed decline and the development of walking speed limitation over the follow-up period. Additionally, whether these associations varied by age, sex, and combined CRFs were further explored.

## Methods

### Study population

**Fig. 1:**
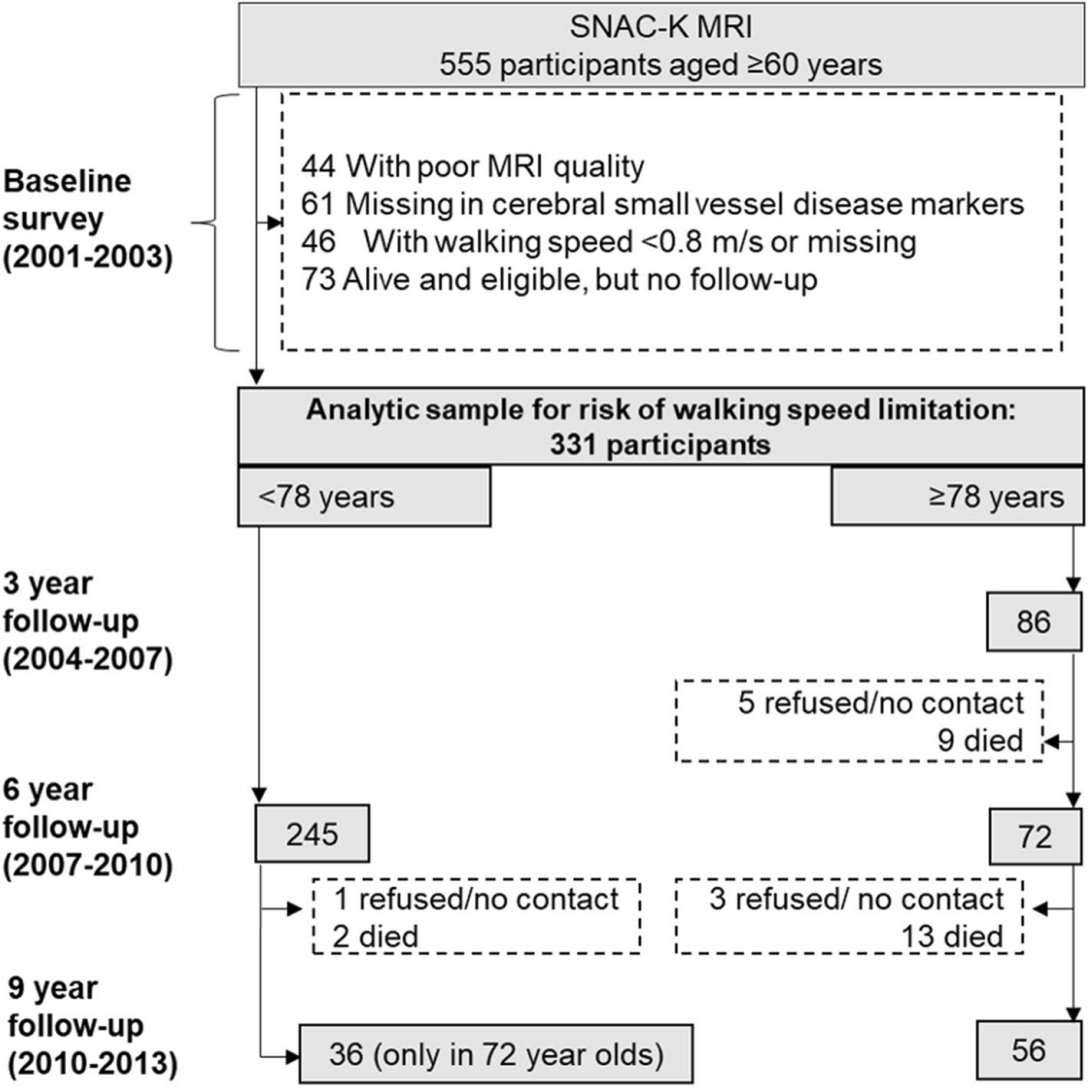
Flowchart of study population (2001-2004 to 2010-2013). Data were collected every six years for the younger-old (60, 66, and 72 years), and every three years for the older-old (≥78 years). The older-old had three follow-up examinations, whereas in the younger-old cohort those 72 years old had two follow-up examinations at six and nine years, and those 60 and 66 years old had only one follow-up examination at six years

Participants were derived from the population-based Swedish National study on Aging and Care in Kungsholmen (SNAC-K), including adults 60 years and older living in their homes or institutions, in a central area of Stockholm, Sweden. Details on study design and data collection can be found elsewhere, www.snac.org [16]. In the SNAC-K study, at baseline (2001-2004), a random sample of eleven age strata was selected, with 6 years between the younger (60, 66, and 72 years old) and 3 years between the older age cohorts (78, 81, 84, 87, 90, and 93 years and older). Follow-up data were collected every 6 years for the younger and every 3 years for the older age cohorts, until 2013 (one examination for the younger cohorts and two examinations for the older cohorts). Data collection was done in this manner due to more rapid changes in health occurring in the older age strata and higher attrition rates. Of the 4590 persons who were alive and eligible, 3363 (73.3%) participated at baseline. This study is based on the brain MRI sub-study embedded in SNAC-K. Participants in this sub-study were recruited at baseline (September 2001 to October 2003), including 555 persons not living in institutions, and who were not disabled nor diagnosed with dementia **(**Fig. 1**)**. Compared with the rest of the SNAC-K sample, those who underwent MRI scans, were significantly younger, more men, higher educated, more physically active, never smokers, non-alcohol drinkers, and with a higher body mass index (BMI). However, there was no significant difference found in regards to presence of cardiovascular risk factors (hypertension, diabetes, and dyslipidaemia) [17].

For this study, those with poor image quality (*n* = 44), missing information on at least one cSVD marker (*n* = 61), with baseline walking speed limitation (< 0.8 m/s) or with missing walking speed information at baseline (*n* = 46), and who were alive and eligible but did not contribute to the follow-up assessments of physical function (*n* = 73), were excluded, leaving an analytical sample of 331 persons. Those who were excluded due to missing data for the current study were older, had a lower level of education, and a higher number of chronic diseases than those included in the analytical sample.

### Ethical approval

The SNAC-K study was performed in accordance with the Declaration of Helsinki and was approved by the Regional Ethics Review Board in Stockholm. Written and verbal informed consent was provided by all participants.

### MRI acquisition and measurements

#### MRI protocol

Participants were scanned on an Intera 1.5 T scanner (Philips Healthcare, Best, the Netherlands). The MRI protocol included three sequences: axial three-dimensional T1-weighted, proton-density/T2-weighted, and axial turbo fluid attenuated inversion recovery (FLAIR). The T1-weighted MRI images were acquired with a 3D fast field echo with the following parameters: repetition time [TR] 15 ms, echo time [TE] 7 ms, flip angle [FA] 15^o^, field of view [FOV] 240, 128 slices with slice thickness 1.5 mm, and in-plane resolution 0.94 × 0.94 mm, no gap, and matrix 256 × 256. The parameters for the a proton-density/T2-weighted sequence were TR 3995 ms, TE 18/90 ms, echo-train length 6, FA 90°, and 60 3-mm sequential images without a gap or angulation. The parameters for FLAIR were TR 6000 ms, TE 100 ms, inversion time 1900 ms, FA 90^o^, echo train length 21, FOV 230, 22 slices with slice thickness 5 mm, in-plane resolution 0.90 × 0.90 mm, gap 1 mm, and matrix 256 × 256 [17–19].

#### MRI markers of cerebral small vessel disease

Three cSVD markers were assessed: WMH, lacunes, and PVS.

1) Global WMH were manually drawn on the FLAIR images by a single rater neuroimaging specialist (G.K.), and further the total volume was calculated after interpolation on the corresponding T1-weighted images to compensate for the gap between slices in FLAIR using MRIcron software. Intra-rater reliability was assessed in a subset of 30 individual data sets 2 years later (randomly chosen from SNAC-K MRI sample), which resulted in a correlation of 0.998 (*P* < 0.001). The Dice coefficient used to determine spatial overlap between two binary images was equal to 0.76 (SD 0.09) for the 30 images, as previously reported [20], which was considered very good agreement between segmentations. T1 images were segmented into grey matter, white matter, and cerebrospinal fluid using SPM12 in MATLAB R2012b. All segments were visually inspected by a neuroimaging expert. WMH volume was adjusted for total brain tissue volume.

2) Lacunes were defined as small lesions with cerebrospinal fluid signal on all sequences and surrounding high signal on FLAIR sequence.

3) PVS were assessed using a visual rating scale, as described in detail earlier [19], where also a description on how to differentiate between lacunes and PVS was explained. Briefly, T1 and T2 images were used to assess PVS in different brain areas (i.e. frontal lobe, parieto-occipital lobe, basal ganglia, including thalami, and subinsular region) in each hemisphere. The number of PVS was scored in each region from 0 to 3: 0 (no visible PVS), 1 (1-5 PVS), 2 (6-10 PVS), or 3 (> 10 PVS), with an excellent intra- and inter-rater reliability (both weighted κ = 0.77). Scores were then summed to a maximum score of 24 [19]. All lacunes and PVS were assessed by a clinical neuroradiologist.

Cerebral SVD burden was estimated using a similar composite measurement that has been previously reported [7], including WMH, PVS, and lacunes. We assigned 1 point to those who were categorised in the top tertile of WMH volume, in the top tertile of the PVS score, or had lacunes. The scores for each of the three cSVD markers that were concurrently present in a participant were then summed to represent the overall cSVD burden, with the score ranging from 0 to 3.

### Assessment of walking speed

Participants were invited to visit the research centre or data collectors visited the participants in their homes when unable to visit the centre. Walking speed (metres per second, m/s) was tested with a nurse present, who asked the participant to walk at a usual pace over 6 or 2.44 m [21], using a stop watch and a 2 m flying start. The shorter distance was used if the participant self-assessed to have a slow usual pace or if the test occurred in their homes where space was restricted. The walking speed test measured with the different distances has been previously reported to be comparable [21], therefore can be used in this manner. Walking speed was examined continuously and dichotomously, with limitation defined according to the well-established cut-off of < 0.8 m/s as previously reported [1, 22, 23].

### Covariates

At baseline, data on potential confounders were collected through interviews, clinical examinations, laboratory tests, and other examinations, by trained staff (physicians, nurses, and psychologists), and retrieved from the Swedish National Patient Register. Demographic data included age, sex, and education (elementary, high school, and university). The modifiable CRFs included physical inactivity, heavy alcohol consumption, ever smoking, hypertension, high total cholesterol, diabetes, and high BMI. Physical inactivity’s assessment and categorisation can be found in detail elsewhere [24]. Briefly, physical activity was assessed through a self-administered questionnaire on frequency and intensity in the last 12 months, which was subsequently categorised as inactive, light, and moderate-to-vigorous intensity. Participants were then divided into active (weekly participation in light or moderate-to-vigorous intensity physical activity) and inactive (never or less than 2-3 times/month) groups [24–27]. The subjective measure of physical activity in the SNAC-K study has been shown to have a moderate level of reliability to accelerometry [24]. Alcohol consumption was categorised into no/occasional/light-to-moderate vs. heavy (> 14 drinks per week for men or > 7 drinks per week for women) [28]. Smoking status was recorded as never or ever. Seated arterial blood pressure was measured twice on the left arm with a sphygmomanometer, and the mean of two measurements was used for the analysis. Systolic blood pressure (SBP) and diastolic blood pressure (DBP) were categorised according to a previous study [29] (SBP: < 130, 130-139, 140-149, and ≥ 150 mmHg; DBP: < 70, 70-79, 80-89, and ≥ 90 mmHg). All medications were classified according to the Anatomical Therapeutic Chemical (ATC) classification system. Hypertension was defined as ≥140/90 mmHg or use of antihypertensive agents (ATC codes C02, C03, and C07-C09). BMI was categorised as underweight (< 20 kg/m^2^), normal (20-24.9), overweight (25-29.9), or obese (≥30). High total cholesterol was defined as a level ≥ 6.22 mmol/l or use of cholesterol lowering agents (ATC code C10). Diabetes was defined according to self-reported history, records from the Swedish National Patient Register, the use of hypoglycaemic agents (ATC code A10), or a glycated haemoglobin level ≥ 6.5% [30].

In addition, C-reactive protein, a marker of inflammation in the body, was measured following a standard protocol, with a high concentration defined as greater than 5 mg/L [31]. Global cognitive function was assessed using the Mini-Mental State Examination (MMSE) administered by a psychologist [32]. Number of chronic diseases (e.g. cancer and osteoporosis) and the presence of cardio- and cerebrovascular diseases (CVDs; i.e. coronary heart disease, atrial fibrillation, heart failure, and cerebrovascular disease) at baseline were defined by integrating information from clinical examination, electrocardiogram, medication use, and the Swedish National Patient Register [33].

### Statistical analysis

Cross-sectional associations between baseline walking speed and the combined and individual cSVD markers were assessed using the general linear model. In order to investigate the average annual change in walking speed (m/s) associated with baseline cSVD markers, linear mixed-effects models were employed using an interaction term between the cSVD markers and follow-up time. Model 1 was unadjusted, whereas model 2 was adjusted for age, sex, education, CRFs, MMSE, number of chronic diseases, CRP, and CVDs. Cox proportional hazards models were performed to investigate the association between individual cSVD markers and their combined burden and incident walking speed limitation, using follow-up time as the time scale. Proportionality was satisfied for all models. Events were censored at first occurrence, otherwise censoring occurred at the end of the follow-up, or when death or dropout occurred. A dummy variable was created for variables with missing information of a covariate (7.2% missing), in order to maximise the sample size. We tested interactions of cSVD markers with age and sex on walking speed limitation in additional Cox models. If the interaction was significant (*P* ≤ 0.05), a subsequent stratified analysis was performed. A CRF score was also calculated by counting the number of the dichotomised CRFs (i.e. hypertension, diabetes, heavy alcohol consumption, physical inactivity, smoking, and high cholesterol). Then, the CRF score was dichotomised based on the median (< 4 or ≥ 4 CRFs) and stratified analyses were performed to test whether the association between each individual cSVD marker on future walking speed limitation and decline varied by combined CRFs, using Cox models and linear mixed-effects models.

Additional sensitivity analyses were performed to determine if death was a competing risk in additional Cox models. Statistical software Stata version 15 (StataCorp, College Station, TX), was used for all analyses. *P* value for significance was set at ≤0.05.

## Results

At baseline, the mean age of the 331 participants was 68.9 (SD 8.3) years and 58.3% were women. Compared with those who did not develop walking speed limitation at follow-up, those who did were more likely to be older, have a lower education and MMSE score, have more chronic diseases, were not smokers, had a higher average SBP, taking anti-hyperintensive agents, and were more likely to have cerebrovascular disease and heart failure (Table 1). At baseline, neither individual nor combined cSVD markers were significantly associated with walking speed (data not shown).

**Table 1:** Baseline characteristics of the total sample and stratification by follow-up walking speed limitation

Characteristics	Total sample (***n*** = 331)	Follow-up walking speed limitation		
		No (***n*** = 255)	Yes (***n*** = 76)	***P*** value
Age (years), mean (SD)	68.9 (8.3)	66.2 (6.8)	77.7 (6.3)	< 0.001
Women, n (%)	193 (58.31)	142 (55.7)	51 (67.1)	0.08
Education, n (%)				0.01
Elementary	32 (9.7)	22 (8.6)	10 (13.2)	
High School	141 (42.6)	100 (39.2)	41 (54.0)	
University	158 (47.7)	133 (52.2)	25 (32.9)	
MMSE score^a^, mean (SD)	29.3 (0.9)	29.3 (0.9)	29.0 (1.1)	0.02
High CRP ^a^, n (%)	54 (16.6)	38 (15.2)	16 (21.1)	0.23
Number of chronic diseases, mean (SD)	3.2 (2.0)	3.0 (1.8)	5.0 (2.2)	< 0.001
Physically inactive, n (%)	54 (16.3)	43 (16.9)	11 (14.5)	0.62
Heavy alcohol consumption, n (%)	65 (19.6)	51 (20.0)	14 (18.4)	0.76
Ever smoking, n (%)	186 (56.2)	152 (59.6)	34 (44.7)	0.02
SBP, mean (SD)	141.4 (19.1)	139.7 (18.6)	147.3 (19.5)	0.002
DBP, mean (SD)	83.2 (10.0)	83.3 (10.0)	82.7 (10.0)	0.62
Anti-hypertensive agents, n (%)	108 (32.6)	68 (26.7)	40 (52.6)	< 0.001
Diabetes, n (%)	22 (6.7)	16 (6.3)	6 (7.9)	0.62
High total cholesterol ^a^, n (%)	192 (58.7)	146 (58.2)	46 (60.5)	0.71
BMI (kg/m^2^), n (%)				0.47
Underweight (< 20)	12 (3.6)	11 (4.3)	1 (1.3)	
Normal (20-24.9)	134 (40.5)	99 (38.8)	35 (46.1)	
Overweight (25-29.9)	147 (44.4)	116 (45.5)	31 (40.8)	
Obese (≥30)	38 (11.5)	29 (11.4)	9 (11.8)	
Atrial fibrillation, n (%)	17 (5.1)	13 (5.1)	4 (5.3)	0.95
Coronary heart disease, n (%)	24 (7.3)	19 (7.5)	5 (6.6)	0.80
Heart failure, n (%)	13 (3.9)	6 (2.4)	7 (9.2)	0.01
Cerebrovascular diseases, n (%)	10 (3.0)	5 (2.0)	5 (6.6)	0.04
WMH volume ^b^, mean (SD)	0.8 (1.3)	0.6 (1.2)	1.3 (1.3)	< 0.001
WMH volume, n (%)				0.001
1st Tertile	118 (35.7)	101 (39.6)	17 (22.4)	
2nd Tertile	126 (38.1)	99 (38.8)	27 (35.5)	
3rd Tertile	87 (26.3)	55 (21.6)	32 (42.1)	
PVS score, mean (SD)	15.6 (4.5)	15.4 (4.6)	16.2 (4.5)	0.20
PVS, n (%)				0.46
1st Tertile	103 (31.1)	82 (32.2)	21 (27.6)	
2nd Tertile	121 (36.6)	95 (37.3)	26 (34.2)	
3rd Tertile	107 (32.3)	78 (30.6)	29 (38.2)	
Presence of lacunes, n (%)	37 (11.2)	22 (8.6)	15 (19.7)	0.01
cSVD burden, n (%)				< 0.001
0	159 (48.0)	138 (54.1)	21 (27.6)	
1	118 (35.7)	82 (32.2)	36 (47.4)	
2 or 3	54 (16.3)	35 (13.7)	19 (25.0)	

^a^ Data were missing for 15 persons in MMSE score, 5 in CRP, and 4 in total cholesterol

^b^ Volume was corrected for total brain tissue volume

*SD* standard deviation, *MMSE* Mini-Mental State Examination, *WMH* white matter hyperintensities, *PVS* perivascular spaces, *CRP* C-reactive protein, *SBP* systolic blood pressure, *DBP* diastolic blood pressure, *BMI* body mass index, *cSVD* cerebral small vessel disease

*P* value is for the test of comparisons between people with and without incident walking speed limitation during the follow-up period

**Fig. 2:**
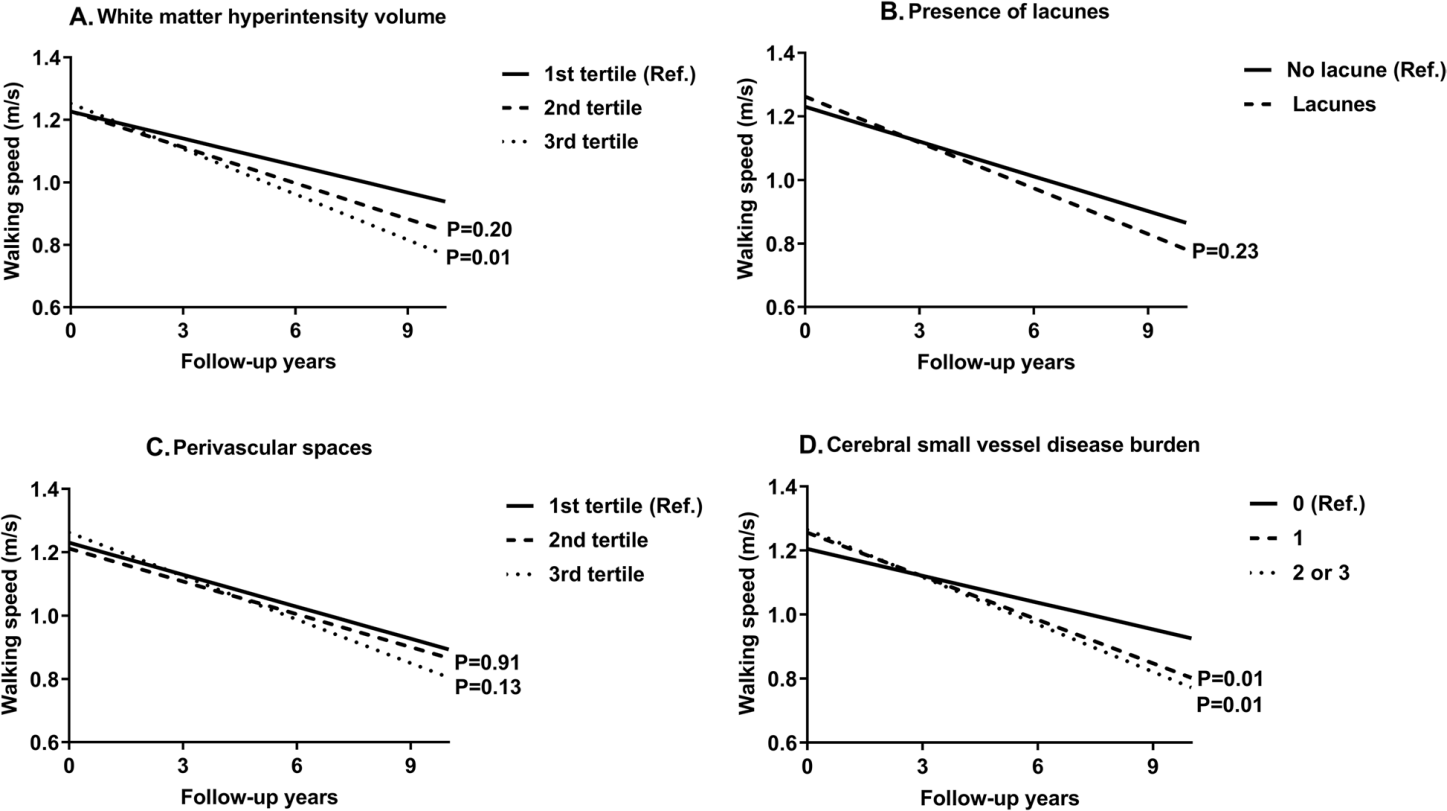
Average annual change in walking speed (m/s) by baseline markers of cerebral small vessel disease. Average annual change in walking speed (m/s) according to baseline (**A**) tertiles of white matter hyperintensity volume; (**B**) the presence of lacunes; (**C**) tertiles of the number of perivascular spaces; and (**D**) cerebral small vessel disease burden. All models were adjusted for age, sex, education, the Mini-Mental State Examination score, cardiovascular risk factors, C-reactive protein, number of chronic diseases, and cardio- and cerebrovascular diseases (*n* = 331)

Linear mixed-effects modelling analysis showed a faster average annual decline in walking speed for those in the third tertile of WMH volume compared with the first tertile (β coefficient − 0.020; 95% confidence interval [CI] -0.035, − 0.004), even after adjusting for socio-demographics, CRFs, CRP, number of chronic diseases, CVDs, and MMSE score **(**Fig. 2**)**. Lacunes and PVS were not significantly associated with walking speed decline. When the three cSVD markers were aggregated into a composite score, the β coefficient (average annual decline) of walking speed was − 0.018 (95% CI -0.030, − 0.004) for having a low cSVD score and − 0.021 (95% CI -0.040, − 0.004) for having a high cSVD score.

During an average of 6.0 (SD 1.4) years of follow-up, 76 persons (23.0%) developed walking speed limitation, with the incidence being 3.83 per 100 person-years. Of these, the average age was 77.7 (SD 6.3) years at baseline and 67.1% were women. The results from the Cox regression analysis showed that after full adjustment, greater WMH volume was significantly associated with a greater risk of developing walking speed limitation (Hazard Ratio [HR] 2.78; 95% CI 1.31-5.89) **(**Table 2). Likewise, an increased risk of incident walking speed limitation was shown for those in the highest tertile of the PVS score, compared with those in the lowest tertile (HR 2.13; 95% CI 1.04-4.36). No statistically significant association was found between lacunes and walking speed limitation. An increasing cSVD score was significantly associated with an increased HR of subsequent walking speed limitation in a dose-dependent manner (*P* for trend = 0.001). The highest cSVD score was associated with an HR of walking speed limitation of 3.78 (95% CI 1.70-8.45).

**Table 2:** Individual and combined cerebral small vessel disease markers and incident walking speed limitation (*n* = 331)

cSVD markers		No. of	No. of	Hazard Ratio (95% Confidence Interval)	
		subjects	cases	Model 1^a^	Model 2^a^
WMH volume, continuous		331	76	1.38 (1.15-1.66)	1.40 (1.11-1.76)
WMH volume	1st Tertile	118	17	1.00 (Ref.)	1.00 (Ref.)
	2nd Tertile	126	27	1.31 (0.71-2.41)	1.70 (0.77-3.79)
	3rd Tertile	87	32	2.35 (1.30-4.25)	2.78 (1.31-5.89)
	*P* for trend			0.003	0.01
Lacunes	No	294	61	1.00 (Ref.)	1.00 (Ref.)
	Yes	37	15	1.81 (1.02-3.21)	1.43 (0.70-2.92)
PVS score, continuous		331	76	1.02 (0.97-1.08)	1.08 (1.01-1.15)
PVS score	1st Tertile	103	21	1.00 (ref.)	1.00 (Ref.)
	2nd Tertile	121	26	0.81 (0.45-1.44)	1.15 (0.57-2.32)
	3rd Tertile	107	29	1.21 (0.69-2.13)	2.13 (1.04-4.36)
	*P* for trend			0.45	0.04
cSVD burden, continuous		331	76	1.62 (1.21-2.16)	1.95 (1.31-2.89)
cSVD burden	0	159	21	1.00 (Ref.)	1.00 (Ref.)
	1	118	36	2.36 (1.38-4.05)	2.34 (1.20-4.58)
	2 or 3	54	19	2.58 (1.38-4.81)	3.78 (1.70-8.45)
	*P* for trend			0.001	0.001

^a^ Model 1: unadjusted; Model 2: adjusted for age, sex, education, the Mini-Mental State Examination score, cardiovascular risk factors (i.e., physical inactivity, heavy alcohol consumption, smoking, hypertension, body mass index, cholesterol, diabetes), C-reactive protein, number of chronic diseases, and cardio- and cerebrovascular diseases

*cSVD* cerebral small vessel disease, *WMH* white matter hyperintensities, *PVS*  perivascular spaces

There were no statistically significant interactions between any of the single cSVD markers and age on incident walking speed limitation. A statistically significant modifying effect of sex (*P* = 0.032) was observed for the association between lacunes and walking speed, such that men with lacunes were more likely to develop walking speed limitation than women in the fully-adjusted models (Men: HR 13.81; 95% CI 1.62-117.99; Women: HR 1.01; 95% CI 0.40-2.56). However, there was no difference in walking speed decline between men and women according to the presence of baseline lacunes. The sensitivity analysis, considering death as a competing event, yielded similar results to the initial analyses (Supplementary Table 1).

**Fig. 3:**
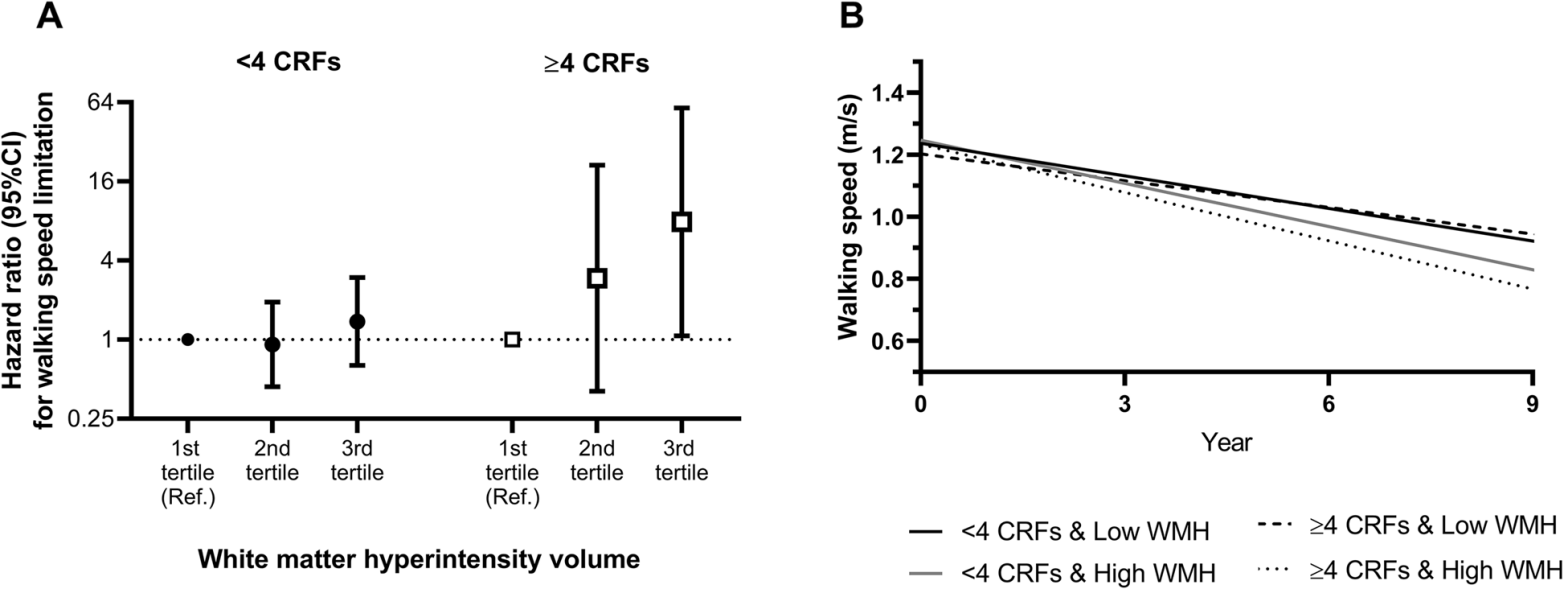
The association between white matter hyperintensity volume and walking speed limitation and decline, by cardiovascular risk factor burden. Association between white matter hyperintensity volume (WMH) and (**A**) future walking speed limitation and (**B**) walking speed decline (m/s) by cardiovascular risk factor burden. All models were adjusted for age, sex, education, the Mini-Mental State Examination score, C-reactive protein, number of chronic diseases, and cardio- and cerebrovascular diseases. CRFs = cardiovascular risk factors; WMH = white matter hyperintensities; CI = confidence interval

The association between WMH volume and walking speed limitation varied by CRF burden, such that those with ≥4 CRFs and were in the highest tertile of WMH volume had a much greater risk for future walking speed limitation (HR 7.87; 95% CI 1.07-57.97) compared with those in the lowest tertile of WMH volume and with ≥4 CRFs (Fig. 3A). In addition, compared with those with low WMH burden and ≥ 4 CRFs, those with high WMH burden and ≥ 4 CRFs indicated a potentially faster decline in walking speed over the follow-up (*P* = 0.06) (Fig. 3B). CRFs did not modify the associations between lacunes or PVS and walking speed.

## Discussion

In this population-based cohort study of older adults without walking speed limitation at baseline, having a greater burden of global cSVD markers simultaneously at baseline was associated with an accelerated decline in walking speed over 9 years of follow-up. Particularly, a greater WMH burden was associated with a faster walking speed decline and future walking speed limitation, independent of demographic factors, cognitive function, chronic conditions, cardio- and cerebrovascular diseases, and other cardiovascular risk factors. A larger number of PVS was also associated with an increased risk of developing walking speed limitation over the follow-up, and lacunes were associated with a higher risk of walking speed limitation only in men. These results not only confirm the association between WMH on the development of poor walking speed, but also demonstrate the potential devastating effects of PVS and lacunes alone and the combined burden of cerebral microvascular abnormalities on age-related changes in walking speed. In addition, having a poor cardiovascular profile together with a high burden of WMH substantially increased the risk of walking speed limitation. Therefore, improvement in modifiable CRFs may help reduce the risk of walking speed limitation associated with WMH.

This is one of the first longitudinal investigations on the association between the burden of multiple cSVD markers and future walking speed among a physically intact older population aged 60 to 90 years. The association between a greater cSVD burden and worse performance on walking speed has been previously reported only in a cross-sectional study [9], where WMH were the main driving cSVD marker. Cerebral SVD burden has also been investigated in association with cognitive decline and dementia in an earlier study from the SNAC-K MRI cohort, revealing that a higher burden of cSVD markers was strongly associated with future cognitive decline and dementia [7]. This may indicate a shared neuropathological basis in the decline of walking speed and cognitive function in older adults.

Population-based cohort studies on the longitudinal associations between the presence of lacunes and walking speed are lacking. Earlier cross-sectional studies have shown inconsistent findings on the association between lacunes and walking speed, with some studies observing an association [12, 34], while others not [9, 35]. In the present study, a significant association was seen only in men. Men are more likely to have a poorer vascular profile than women, which may account for a more advanced vascular pathology. The men in the present study were more likely to have diabetes, be obese, and have cardiovascular disease compared with the women (Supplementary Table 2). However, the association between lacunes and walking speed was present after adjusting for CRFs. Thus, other covert pathological pathways may be at play such as cerebral blood flow and glucose metabolism. Furthermore, enlarged PVS are increasingly being recognised as a marker of cSVD and have been associated with cognitive decline in healthy older adults [19, 36], but cross-sectional studies have exhibited no correlation between PVS and walking speed [9, 35]. Thus, a high burden of PVS may be more correlated with cognitive than physical decline, but may indicate risk of incident walking speed limitation, as observed in the present study.

The strong association observed between cSVD markers and walking speed decline supports vascular brain injury to be a neuropathological basis for physical function diminishment in old age. This in turn reflects the robustness of walking speed as a predictor of many adverse health outcomes [1]. Walking speed is a known superior, simple tool of physical function, and therefore, can be considered as a reliable substitute in clinical and general population settings where a full physical function battery of tests is not feasible to implement. Walking speed assesses not only the physical health of older adults, but also underlying pathologies in the absence of overt conditions. Therefore, using a simple and valid walking speed tool, as in the present study, can reduce administrative burden and increase feasibility for use in more ecologically valid settings.

A potential underlying mechanism in which a greater WMH burden may promote the decline in walking speed is through changes in white matter microstructural integrity. Emerging evidence suggests that greater microstructural integrity of normal-appearing white matter may be a key factor in the preservation of physical function [10]. One study of community-dwelling older adults found that those with a high WMH burden and low microstructural white matter integrity had slower walking speeds [10]. Other mechanisms may be pertaining to the “disconnection syndrome”. This theory suggests that WMH compromise sensorimotor function by disrupting the complex neural networks in cortical regions with projection and association fibres involved in motor control, especially in the frontal regions [37, 38]. Furthermore, slow walkers have been observed to also have reduced neurovascular coupling. However, older adults with WMH who demonstrate effective alignment of cerebral blood flow with cognitively demanding tasks indicated a better preservation of walking speed [39]. Moreover, it is known that older adults recruit more brain regions when undertaking motor tasks than young people, and that WMH accrual may heighten the decline in neural plasticity and lead to physical impairment [37]. More studies are required to disentangle the specific brain regions involved in the decline of walking speed. Additionally, CRFs are known to be strongly associated with cSVD markers and a high CRF burden may exacerbate the association between WMH and walking speed limitation. Thus, improvements on modifiable CRFs may help in slowing the decline in walking speed due to cSVD. In fact, a multi-domain intervention targeting several CRFs simultaneously found improvements in cognitive performance over 2 years in older adults [40]. In addition, a randomised controlled trial including persons with early Alzheimer’s disease found that a lifestyle intervention aimed at improving CRFs, such as BMI, physical inactivity, smoking, and hypertension, slowed the progression of WMH compared with those who received usual care [41]. Thus, these modifiable lifestyle factors may admittedly be potential targets for decreasing the risk of future walking speed impairment among older adults.

Strengths of our study are the prospective design and the population-based cohort of older adults, as well as the objective measure of walking speed. However, our study was not without limitations. The lack of additional cSVD markers such as cerebral microbleeds and microinfarcts may have underestimated the association of a burden of cSVD with physical dysfunction. These additional cSVD markers were not included in the present study due to the lack of respective MRI sequences in the SNAC-K MRI study. Some of the CRFs were self-reported, which could lead to an overestimation, particularly for physical activity. Underestimation may have occurred due to dropouts, owing to their greater likelihood of having more cSVD and poorer physical function. In addition, the data were limited by only having MRI markers and CRFs available at baseline. Also, exclusion of people with walking speed limitation at baseline might affect our results on walking speed. However, the sensitivity analysis including persons with walking speed limitation at baseline yielded similar results to the main analysis (Supplementary Fig. 1). Finally, caution is needed when generalising our findings to other populations due to the high socioeconomic position of our study sample.

## Conclusions

This population-based cohort study showed that a high burden of cSVD markers is associated with accelerated walking speed decline and an increased risk of walking speed limitation among older adults. Additionally, improving CRFs may help in mitigating the negative effects of WMH on walking speed. This provides greater understanding into the potential neuropathological pathways underpinning the decline in walking speed in older adults, and thus, paves the way for the development of potential interventions to help older adults maintain functional independence into later life.

## Supplementary Information

**Additional file 1: Supplementary Table 1.** Competing risk analysis. **Supplementary Table 2.** Baseline characteristics of the total sample and stratification by sex. **Supplementary Fig. 1.** Average annual change in walking speed by baseline markers of cerebral small vessel disease including individuals with walking speed limitation at baseline.

## Data Availability

The datasets during the current study are not publicly available, but are available upon reasonable request through the SNAC-K website: https://www.snac-k.se/for-researchers/application-form/.
