# Supplementary material for: Cerebral small vessel disease, cardiovascular risk factors, and future walking speed in old age: a population-based cohort study
Source: BMC Neurol. 2021 Dec 24;21:496. doi: 10.1186/s12883-021-02529-6 (PMC8705459; doi:10.1186/s12883-021-02529-6)
Supplement: Supplementary file 1 — Additional file 1: Supplementary Table 1. Competing risk analysis. Supplementary Table 2. Baseline characteristics of the total sample and stratification by sex. Supplementary Fig. 1. Average annual change in walking speed by baseline markers of cerebral small vessel disease including individuals with walking speed limitation at baseline. [file 12883_2021_2529_MOESM1_ESM.docx]

**Cerebral small vessel disease, cardiovascular risk factors, and future walking speed in old age: a population-based cohort study**

Emerald G. Heiland ^a,b,c^, PhD; Anna-Karin Welmer ^b,d,e,f^, PhD; Grégoria Kalpouzos ^b^, PhD; Anna Laveskog ^g,h^, MD; Rui Wang ^b,c^, PhD; Chengxuan Qiu ^b^, PhD

^a^ Department of Surgical Sciences, Medical Epidemiology, Uppsala University, Dag Hammarskjölds väg 14B, 75 185 Uppsala, Sweden

^b^ Aging Research Center, Department of Neurobiology, Care Sciences and Society, Karolinska Institutet-Stockholm University, Tomtebodavägen 18A, 171 65 Solna, Sweden

^c^ Department of Physical Activity and Health, The Swedish School of Sport and Health Sciences (GIH), Lidingövägen 1, 114 86 Stockholm, Sweden

^d^ Stockholm Gerontology Research Center, Sveavägen 155, 113 46 Stockholm, Sweden

^e^ Women´s Health and Allied Health Professionals Theme, Medical Unit Medical Psychology, Karolinska University Hospital, Stockholm, Sweden

^f^ Division of Physiotherapy, Department of Neurobiology, Care Sciences and Society, Karolinska Institutet, Alfred Nobels allé 23, 141 83 Huddinge, Sweden.

^g^ Division of Neuro, Department of Clinical Neuroscience, Karolinska Institutet, Tomtebodavägen 18A, 171 65 Solna, Sweden

^h^ Department of Neuroradiology, Karolinska University Hospital, Eugeniavägen 3, 171 76 Solna, Sweden

Corresponding Author:

Emerald G. Heiland, Department of Surgical Sciences, Medical Epidemiology, Uppsala University, Dag Hammarskjölds väg 14B, 75 185 Uppsala, Sweden, Email: [emerald.heiland@surgsci.uu.se](mailto:emerald.heiland@surgsci.uu.se) or Chengxuan Qiu, Aging Research Center, Karolinska Institutet, Widerströmska Huset, [Tomtebodavägen 18A](https://email.ki.se/owa/redir.aspx?C=JmJMFVoVlMUkkb2hytTomG5oxCp-D_DHJBGfr_PJ2djl_3m8IFrWCA..&URL=https%3a%2f%2furldefense.proofpoint.com%2fv2%2furl%3fu%3dhttps-3A__maps.google.com_-3Fq-3DTomtebodav-25C3-25A4gen-2B18A-2B-250D-250A-2BSE-2D171-2B65-2BSolna-2B-250D-250A-2BSWEDEN-26entry-3Dgmail-26source-3Dg%26d%3dDwMF-g%26c%3d4J0DyIxNDQjAR-x4NjL0NpGXBwDO_RhvZ8C7KRkAL-I%26r%3dbJ1aoX8Bl_X7zSzEV_OGJ0aqI2BWOBuS8qFBLEpd1MU%26m%3da9T0axXVfWcngDpCLHQ6fv3fWVgBjlWw8Mxd1b_QjTo%26s%3dYESqfQksikXnVezDjDdrzyYBBH3RNoAOyRvIpJO6zrI%26e%3d), [171](https://email.ki.se/owa/redir.aspx?C=JmJMFVoVlMUkkb2hytTomG5oxCp-D_DHJBGfr_PJ2djl_3m8IFrWCA..&URL=https%3a%2f%2furldefense.proofpoint.com%2fv2%2furl%3fu%3dhttps-3A__maps.google.com_-3Fq-3DTomtebodav-25C3-25A4gen-2B18A-2B-250D-250A-2BSE-2D171-2B65-2BSolna-2B-250D-250A-2BSWEDEN-26entry-3Dgmail-26source-3Dg%26d%3dDwMF-g%26c%3d4J0DyIxNDQjAR-x4NjL0NpGXBwDO_RhvZ8C7KRkAL-I%26r%3dbJ1aoX8Bl_X7zSzEV_OGJ0aqI2BWOBuS8qFBLEpd1MU%26m%3da9T0axXVfWcngDpCLHQ6fv3fWVgBjlWw8Mxd1b_QjTo%26s%3dYESqfQksikXnVezDjDdrzyYBBH3RNoAOyRvIpJO6zrI%26e%3d) [65 Solna](https://email.ki.se/owa/redir.aspx?C=JmJMFVoVlMUkkb2hytTomG5oxCp-D_DHJBGfr_PJ2djl_3m8IFrWCA..&URL=https%3a%2f%2furldefense.proofpoint.com%2fv2%2furl%3fu%3dhttps-3A__maps.google.com_-3Fq-3DTomtebodav-25C3-25A4gen-2B18A-2B-250D-250A-2BSE-2D171-2B65-2BSolna-2B-250D-250A-2BSWEDEN-26entry-3Dgmail-26source-3Dg%26d%3dDwMF-g%26c%3d4J0DyIxNDQjAR-x4NjL0NpGXBwDO_RhvZ8C7KRkAL-I%26r%3dbJ1aoX8Bl_X7zSzEV_OGJ0aqI2BWOBuS8qFBLEpd1MU%26m%3da9T0axXVfWcngDpCLHQ6fv3fWVgBjlWw8Mxd1b_QjTo%26s%3dYESqfQksikXnVezDjDdrzyYBBH3RNoAOyRvIpJO6zrI%26e%3d), Sweden. Tel.: +46 852485821, Email: [chengxuan.qiu@ki.se](mailto:chengxuan.qiu@ki.se).

**Supplementary Table 1.** Hazard ratios (95% confidence intervals) of incident walking speed limitation associated with individual markers of cerebral small vessel disease and their burden, from the competing risk analysis taking death into consideration (n=331).

| Cerebral SVD markers | | No. of subjects | No. of cases | Hazard Ratio (95% Confidence Interval) |
| --- | --- | --- | --- | --- |
| WMH volume, continuous | |  |  | 1.33 (1.05-1.68) |
| WMH volume | 1^st^ Tertile | 118 | 17 | 1.00 (Ref.) |
|  | 2^nd^ Tertile | 126 | 27 | 1.50(0.65-3.48) |
|  | 3^rd^ Tertile | 87 | 32 | 2.12 (0.98-4.56) |
|  | *P* for trend |  |  | 0.05 |
| Lacunes | No | 294 | 61 | 1.00 (Ref.) |
|  | Yes | 37 | 15 | 1.35 (0.64-2.83) |
| PVS score, continuous | |  |  | 1.06 (0.99-1.14) |
| PVS score | 1^st^ Tertile | 103 | 21 | 1.00 (Ref.) |
|  | 2^nd^ Tertile | 121 | 26 | 1.05 (0.48-2.28) |
|  | 3^rd^ Tertile | 107 | 29 | 1.85 (0.90-3.81) |
|  | *P* for trend |  |  | 0.09 |
| SVD burden, continuous |  |  |  | 1.67 (1.13-2.48) |
| SVD burden | 0 | 159 | 21 | 1.00 (Ref.) |
|  | 1 | 118 | 36 | 1.93 (0.99-3.75) |
|  | 2 or 3 | 54 | 19 | 2.67 (1.21-5.89) |
|  | *P* for trend |  |  | 0.01 |
| Adjusted for age, sex, education, Mini-Mental State Examination score, cardiovascular risk factors (i.e., physical inactivity, heavy alcohol consumption, smoking, hypertension, body mass index, cholesterol, diabetes), C-reactive protein, number of chronic diseases, and cardiovascular diseases.  SVD=cerebral small vessel disease; WMH=white matter hyperintensities; PVS=perivascular spaces. | | | | |

| **Supplementary Table 2.** Baseline characteristics of the total sample and stratification by sex. | | | | |
| --- | --- | --- | --- | --- |
| **Characteristics** | **Total sample**  **(n=331)** | **Sex** | | |
|  |  | **Men**  **(n=138)** | **Women**  **(n=193)** | **P value** |
| Age (years), mean (SD) | 68.9 (8.3) | 69.0 (8.7) | 68.8 (8.0) | 0.808 |
| Education, n (%) |  |  |  | 0.001 |
| Elementary | 32 (9.7) | 14 (10.1) | 18 (9.3) |  |
| High School | 141 (42.6) | 43 (31.2) | 98 (50.8) |  |
| University | 158 (47.7) | 81 (58.7) | 77 (39.9) |  |
| MMSE score^a^, mean (SD) | 29.3 (0.9) | 29.3 (1.0) | 29.3 (0.9) | 0.911 |
| High CRP ^a^, n (%) | 54 (16.6) | 22 (16.3) | 32 (16.8) | 0.913 |
| Number of chronic diseases, mean (SD) | 3.2 (2.0) | 3.2 (2.2) | 3.2 (1.9) | 0.863 |
| Physically inactive, n (%) | 54 (16.3) | 25 (18.1) | 29 (15.0) | 0.453 |
| Heavy alcohol consumption, n (%) | 65 (19.6) | 14 (10.1) | 51 (26.4) | <0.001 |
| Ever smoking, n (%) | 186 (56.2) | 83 (60.1) | 103 (53.4) | 0.22 |
| SBP, mean (SD) | 141.4 (19.1) | 142.3 (18.4) | 140.8 (19.5) | 0.465 |
| DBP, mean (SD) | 83.2 (10.0) | 83.8 (10.4) | 82.7 (9.7) | 0.332 |
| Anti-hypertensive agents, n (%) | 108 (32.6) | 50 (36.2) | 58 (30.1) | 0.237 |
| Diabetes, n (%) | 22 (6.7) | 17 (12.3) | 5 (2.6) | <0.001 |
| High total cholesterol ^a^, n (%) | 192 (58.7) | 78 (57.8) | 114 (59.4) | 0.773 |
| BMI (kg/m^2^), n (%) |  |  |  | 0.060 |
| Underweight (<20) | 12 (3.6) | 1 (0.7) | 11 (5.7) |  |
| Normal (20-24.9) | 134 (40.5) | 60 (43.5) | 74 (38.3) |  |
| Overweight (25-29.9) | 147 (44.4) | 58 (42.0) | 89 (46.1) |  |
| Obese (≥30) | 38 (11.5) | 19 (13.8) | 19 (9.8) |  |
| Atrial fibrillation, n (%) | 17 (5.1) | 12 (8.7) | 5 (2.6) | 0.013 |
| Coronary heart disease, n (%) | 24 (7.3) | 20 (14.5) | 4 (2.1) | <0.001 |
| Heart failure, n (%) | 13 (3.9) | 8 (5.8) | 5 (2.6) | 0.139 |
| Cerebrovascular diseases, n (%) | 10 (3.0) | 4 (2.9) | 6 (3.1) | 0.912 |
| WMH volume, mean (SD) ^b^ | 0.8 (1.3) | 1.0 (1.2) | 0.6 (1.3) | 0.014 |
| WMH volume, n (%) |  |  |  |  |
| 1^st^ Tertile | 118 (35.7) | 37 (26.8) | 81 (42.0) | 0.015 |
| 2^nd^ Tertile | 126 (38.1) | 62 (44.9) | 64 (33.2) |  |
| 3^rd^ Tertile | 87 (26.3) | 39 (28.3) | 48 (24.9) |  |
| PVS score, mean (SD) | 15.6 (4.5) | 15.8 (4.6) | 15.4 (4.5) | 0.410 |
| PVS, n (%) |  |  |  |  |
| 1^st^ Tertile | 103 (31.1) | 39 (28.3) | 64 (33.2) | 0.632 |
| 2^nd^ Tertile | 121 (36.6) | 53 (38.4) | 68 (35.2) |  |
| 3^rd^ Tertile | 107 (32.3) | 46 (33.3) | 61 (31.6) |  |
| Presence of lacunes, n (%) | 37 (11.2) | 17 (12.3) | 20 (10.4) | 0.578 |
| cSVD burden, n (%) |  |  |  |  |
| 0 | 159 (48.0) | 64 (46.4) | 95 (49.2) | 0.738 |
| 1 | 118 (35.7) | 49 (35.5) | 69 (35.8) |  |
| 2 or 3 | 54 (16.3) | 25 (18.1) | 29 (15.0) |  |
| ^a^ Data were missing for 15 persons in MMSE score, 5 in CRP, and 4 in total cholesterol.  ^b^ Volume was corrected for total brain tissue volume.  SD=standard deviation; MMSE=Mini-Mental State Examination; WMH=white matter hyperintensities; PVS=perivascular spaces; CRP=C-reactive protein; SBP=systolic blood pressure; DBP=diastolic blood pressure; BMI=body mass index; cSVD=cerebral small vessel disease.  *P* value is for the test of comparisons between men and women. | | | | |


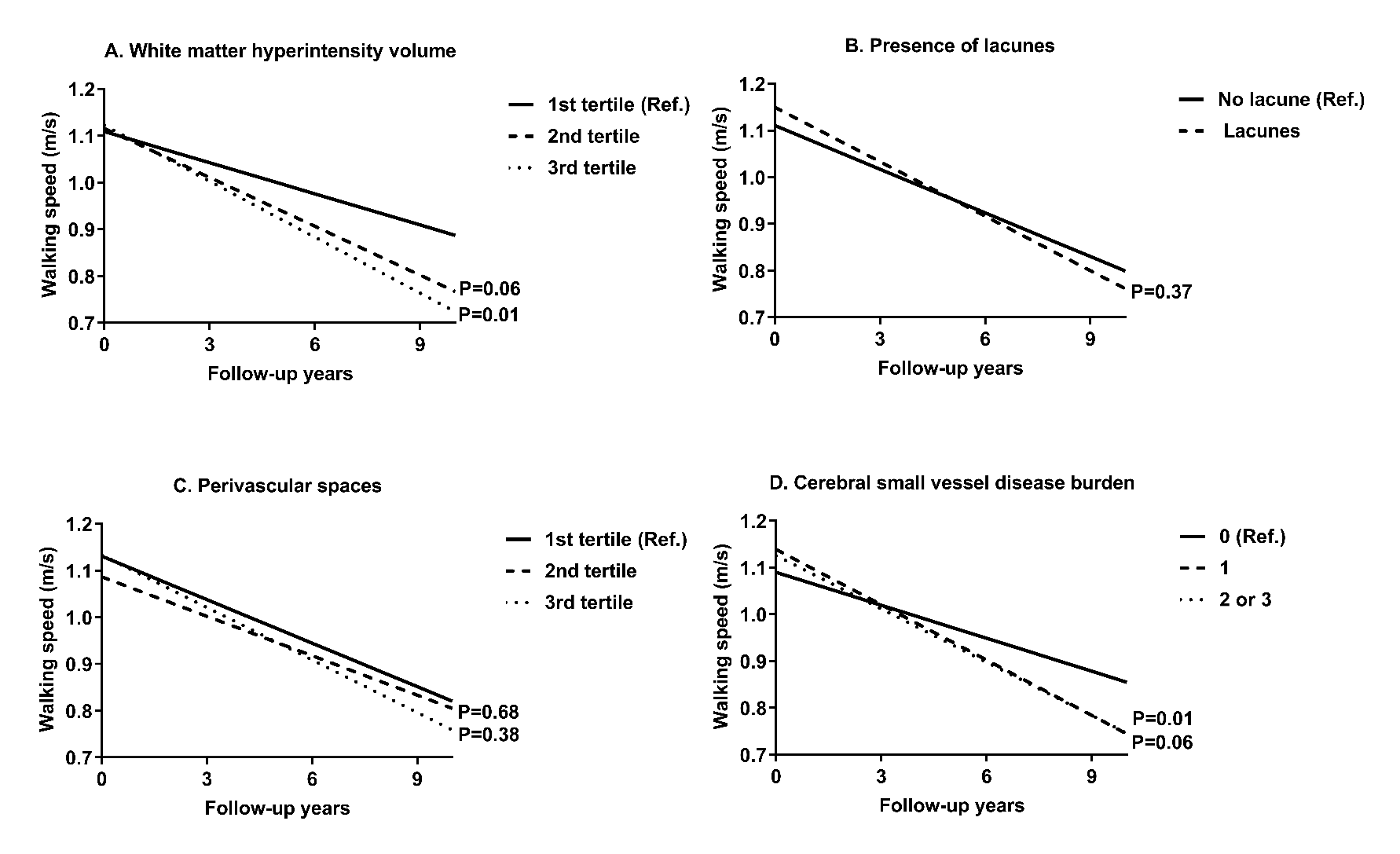
 **Supplementary Figure 1.** *Average annual change in walking speed (m/s) by baseline markers of cerebral small vessel disease including individuals with walking speed limitation at baseline.*

Average annual change in walking speed (m/s) according to baseline (A) tertiles of white matter hyperintensity volume; (B) the presence of lacunes; (C) tertiles of the number of perivascular spaces; and (D) cerebral small vessel disease burden. All models were adjusted for age, sex, education, the Mini-Mental State Examination score, cardiovascular risk factors, number of chronic diseases, C-reactive protein, and cardio- and cerebrovascular diseases (n=450).
